# Implant-free loop tenodesis compared to arthroscopic anchor tenodesis for the treatment of long head of biceps tendon disorders (LOOPTEN trial): study protocol for a multi-center non-inferiority randomized controlled trial

**DOI:** 10.1186/s12891-025-08818-2

**Published:** 2025-06-06

**Authors:** Leopold Henssler, Florian Zeman, Doruk Akgün, Kathi Thiele, Stephan Pauly, Stefan Greiner, Andreas Voss, Christian Gerhardt, Lars-Johannes Lehmann, Gunnar Huppertz, Magdalena Elsner, Volker Alt, Michael Koller, Maximilian Kerschbaum

**Affiliations:** 1 Department of Trauma Surgery, University Hospital Regensburg, Franz-Josef-Strauss-Allee 11, Regensburg, 93053 Germany; 2 Center for Clinical Studies, University Hospital Regensburg, Franz-Josef-Strauss-Allee 11, Regensburg, 93053 Germany; 3 Center for Musculoskeletal Surgery, Charité University Medicine, Campus Virchow-Klinikum, Mittelallee 3, Berlin, 13353 Germany; 4 Department for Shoulder Surgery, Vivantes Auguste-Viktoria-Clinic, Rubensstraße 125, Berlin, 12157 Germany; 5 Sporthopaedicum Regensburg, Straubinger Str. 30, Regensburg, 93055 Germany; 6 Department of Trauma Surgery, Hand Surgery and Sports Medicine, ViDia Clinic Karlsruhe, Steinhäuserstraße 18, Karlsruhe, 76135 Germany

**Keywords:** Arthroscopy, Shoulder, Rotator cuff, Labrum, Biceps, Tendon, Tenodesis, Tenotomy

## Abstract

**Background:**

Pathologies of the long head of the biceps (LHB) tendon are frequently seen as concomitant pathologies during arthroscopic surgery for rotator cuff injuries or the labroligamentous complex of the shoulder. Currently, there are two treatment options: Tenotomy is quick and easy to perform with low complications rates, but has limited functional results, especially in demanding patients; tenodesis of the tendon has shown beneficial cosmetic and functional results, but usually requires an implant for tendon-to-bone attachment and, therefore, carries the risk of implant-related complications. The implant-free loop tenodesis (LT) procedure was developed to combine the advantages of both treatment modalities and has shown promising functional and cosmetic results in a prospective pilot study. This study aims to establish the implant-free LT procedure versus arthroscopic anchor tenodesis (AAT) for the treatment of LHB pathologies during shoulder arthroscopy in terms of structural and functional outcome.

**Methods:**

A national multi-center, two-arm, parallel-group, randomized, controlled, non-inferiority trial will be conducted. Patients are eligible for trial participation if they are at least 18 years of age and present to one of the five enrolling centers with LHB tendon-associated complaints and MRI-confirmed LHB tendinopathy, instability due to SLAP or pulley lesions, or partial rupture. Patients with current or previous shoulder injury that would interfere with post-treatment rehabilitation or study assessment will be excluded from study participation. Participating patients will be randomized 1:1 to receive either LT or AAT and will be followed up for 24 months after surgery. The primary endpoint will be the functional and cosmetic outcome as assessed by the biceps-specific LHB score at 12 months after surgery. Secondary outcomes include assessment of surgery-related complications, overall shoulder and arm function, and structural outcome as evaluated by ultrasound and an additional MRI scan at the final study visit.

**Discussion:**

The study will evaluate whether the implant-free loop tenodesis procedure is non-inferior to arthroscopic implant-based tenodesis in terms of functional and cosmetic results at 12 months post-treatment.

**Trial registration:**

Trial was prospectively registered at the German Clinical Trials Register (DRKS) on 12^th^ June 2024, Registration-ID DRKS00034361, https://drks.de/search/de/trial/DRKS00034361.

## Administrative information

Note: the numbers in curly brackets in this protocol refer to SPIRIT checklist item numbers. The order of the items has been modified to group similar items (see http://www.equator-network.org/reporting-guidelines/spirit-2013-statement-defining-standard-protocol-items-for-clinical-trials/).

**Table:** 

Title	Implant-free loop tenodesis compared to arthroscopic anchor tenodesis for the treatment of long head of biceps tendon disorders (LOOPTEN trial): study protocol for a multi-center non-inferiority randomized controlled trial
Trial registration	German Clinical Trials Register (DRKS), registered 12^th^ June 2024, Registration-ID DRKS00034361, https://drks.de/search/de/trial/DRKS00034361
Protocol version	Version 1.2 (December 2024)
Funding	German Federal Ministry of Education and Research (BMBF), grant number (Förderkennzeichen) 01 KG2404
Author details	[see above]
Name and contact information for the trial sponsor	University Hospital Regensburg 93042 Regensburg Germany
Role of sponsor	Neither the study sponsor nor any funders are involved in study design, data collection, data management, data analysis, interpretation of data, writing of the report or the decision to submit the report for publication. The authority over any of these activities is restricted to the LOOPTEN collaborators.

## Introduction

### Background and rationale 

In the adult patient population, shoulder pain is commonly associated with pathologies of the long head of biceps (LHB) tendon [1]. While shoulder arthroscopy is the fourth most common procedure performed by orthopedic surgeons in the United States [2], up to 75% of these surgical procedures are associated with concomitant pathologies of the LHB tendon [3], which should be treated concomitantly to avoid revision surgery [4]. Besides, the number of arthroscopic LHB tenodesis procedures has steadily increased over the past decade as the field of indications has widened [5].

The benefits of tenodesis versus tenotomy for the treatment of the long head of the biceps tendon have been investigated in the past. A systematic review and meta-analysis that analyzed only RCTs and prospective cohort studies showed a threefold increased risk of Popeye deformity after arthroscopic tenotomy versus tenodesis [6]. Moreover, worse functional outcomes and a higher risk of cramping pain were observed after tenotomy [6]. The functional advantages and decreased rates of Popeye deformity after tenodesis compared to tenotomy have been confirmed in several meta-analyses [7, 8], but these analyses only included studies in which LHB pathologies were treated concomitantly with rotator cuff repair, which is a much more common setting than single treatment of the LHB tendon. Therefore, tenodesis is currently considered the gold standard for treating LHB tendon disorders. However, there are numerous tenodesis techniques that differ in the way the LHB tendon is reattached to bone or soft tissue and the location of reattachment. When comparing arthroscopic suprapectoral tenodesis procedures to open subpectoral techniques, systematic reviews and meta-analyses have reported a higher complication rate for open subpectoral tenodesis [9], although both treatments showed no significant difference in functional outcomes [9, 10]. Additionally, implant-associated complications have been reported regardless of the fixation location, including implant failure [11], increased rates of wound and implant infection [12, 13], and implant irritation [14]. These complications have contributed to a higher revision rate for implant-based LHB tenodesis compared to soft-tissue tenodesis [12].

As an implant-free alternative, the “loop tenodesis” technique [15] has shown encouraging results in preliminary studies [16, 17] and is, therefore, already gaining popularity among international shoulder surgeons. The technique was introduced in 2019 and was driven by the idea of applying an easy and quick to perform technique without the need for an implant and providing stable fixation comparable to standard tenodesis techniques [15]. The principle of the technique is to create a tendon loop at the tendon stump after tenotomy (hence the term “loop tenodesis”). This loop prevents the proximal tendon from slipping through the bicipital groove upon release. As a result, the tendon loop autotenodeses in the surrounding tissue within the bicipital groove through scar formation. After a successful biomechanical study proving significantly higher stability compared to tenotomy [16], loop tenodesis was implemented in a prospective cohort pilot study that showed good short-term improvement in biceps and overall shoulder function [17]. Nevertheless, a high-evidence study directly comparing the new implant-free loop tenodesis procedure with the current standard of implant-based tenodesis technique has been missing so far.

### Objectives 

The objective of the presented study is to compare the implant-free loop tenodesis technique (experimental intervention) with the arthroscopic implant-based suprapectoral tenodesis technique (standard-of-care intervention) in a randomized, controlled, and patient- and outcome-assessor-blinded trial. The aim is to investigate the non-inferiority of the implant-free loop tenodesis technique in terms of the primary endpoint of functional outcome (LHB score) at 12 postoperative months.

Additionally, the safety of both procedures will be investigated as an additional outcome parameter of primary concern. Secondary endpoints will be functional (LHB score, Constant score, ASES score, DASH score, and SF-36) and structural outcome of the tenodesis procedures over a two-year follow-up.

### Trial design

The trial is designed as a multi-center, randomized, controlled, patient- and outcome-assessor-blinded, non-inferiority clinical trial with 1:1 allocation to two parallel groups. The total follow-up period will be 24 months after surgery. Participation in the trial is voluntary, and patients may withdraw their informed consent at any time without compromising their high-quality care. The protocol conforms to the Standard Protocol Items: Recommendations for Interventional Trials (SPIRIT) guideline.

## Methods: participants, interventions and outcomes

### Study setting

The trial will be conducted at five German medical centers with high expertise in shoulder surgery. All investigators are shoulder surgeons certified by the German, Austrian, and Swiss Society for Shoulder and Elbow Surgery (DVSE) and renowned experts in the field. In preparation of the trial, participating centers had to provide the average number of potentially eligible patients using hospital data management systems and ICD-10 codes of the included pathologies (M75.2, M75.6, S43.4, S46.1, S46.2) over the past 5 years to ensure access to the relevant target population and site capability. Capability of recruitment and recruitment progress by predefined milestones will be independently monitored by a CRO for all centers. All investigators and observers at each study site were trained in ICH-GCP E6 (R2) guidelines prior to study initiation.

The following centers met the selection criteria and have agreed to participate in the conduct of the trial: Department of Trauma Surgery, University Hospital Regensburg; Center for Musculoskeletal Surgery, Charité Berlin; Department of Shoulder Surgery, Vivantes Auguste-Viktoria-Clinic Berlin; Department of Trauma Surgery, Hand Surgery and Sports Medicine, St. Vincentius Clinic Karlsruhe; Sporthopeadicum Regensburg/Straubing.

#### Trial flow

**Fig. 1:**
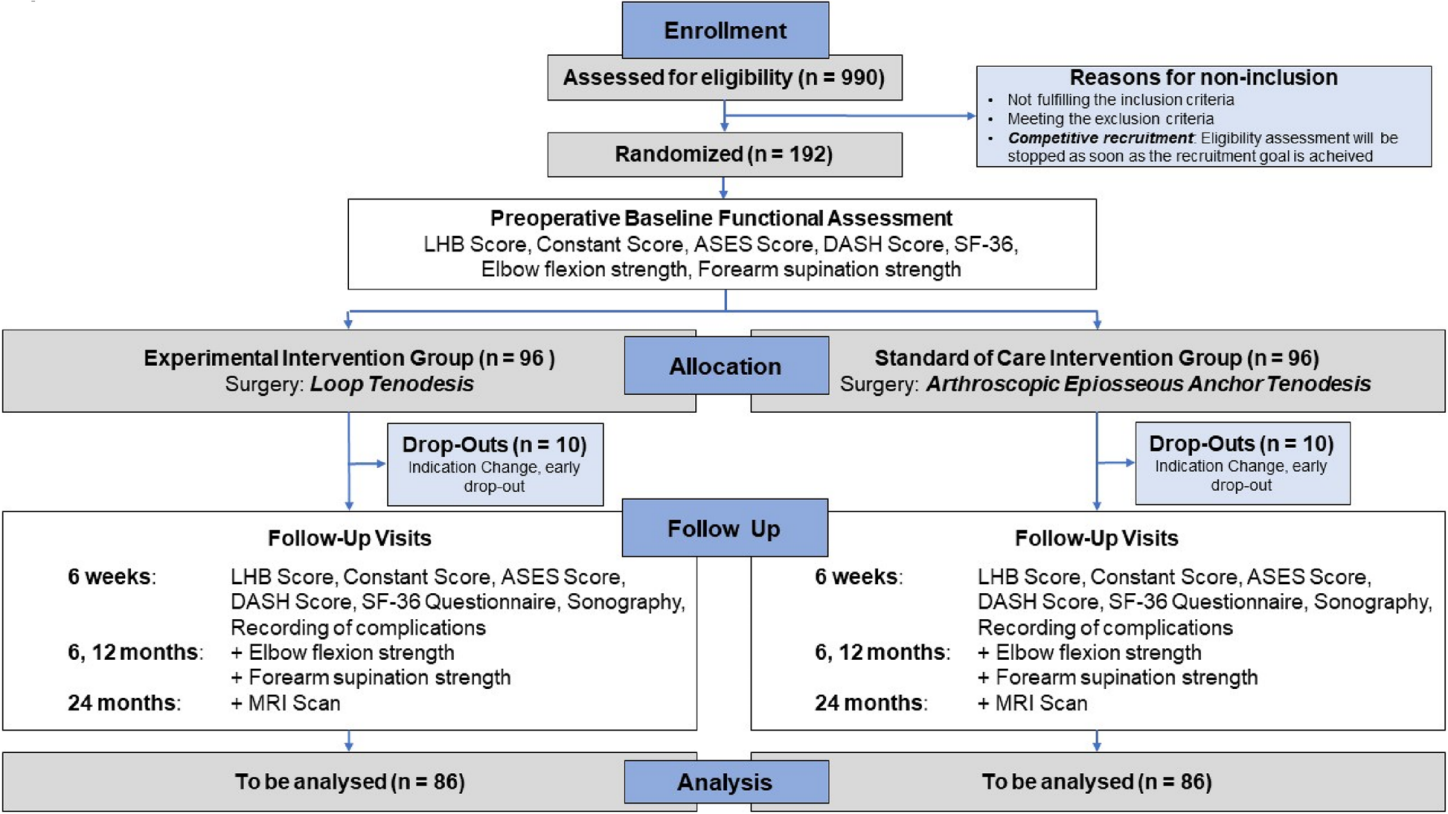
Trial flow of LOOPTEN randomized controlled trial

One hundred ninety-two patients (*n* = 96 for experimental group, *n* = 96 for standard-of-care group) with LHB tendon disorders meeting all eligibility criteria will be randomized equally to implant-free loop tenodesis or arthroscopic epiosseous anchor tenodesis of the LHB tendon. The overall trial flow and timeline for data collection is outlined in Fig. 1.

### Eligibility criteria 

Patients presenting at one of the recruiting centers will be considered for study inclusion, if they:

1. Are at least 18 years of age at the time of presentation,
2. Are able to speak and read German in order to understand the information given and be able to complete the study paperwork,
3. Give written informed consent,
4. Present with at least one of the following pathologies of the long head of the biceps tendon that are considered the most common indications for biceps tenodesis according to Creech et al. [18]:
  - partial or subtotal tears of the LHB tendon,
  - chronic LHB tendon inflammation,
  - LHB tendon instability (pulley lesions),
  - traumatic or degenerative lesions of the LHB tendon insertion at the superior labrum (SLAP lesion).

To ensure unrestricted implementation of the postoperative follow-up treatment, allow for adequate assessment during study visits, and guarantee unbiased study data, patients presenting with at least one of the following conditions will have to be excluded:

1. osteoarthritis of the affected or contralateral shoulder
2. shoulder stiffness
3. chronic glenohumeral instability
4. (previous) distal biceps tendon lesions
5. (previous) surgery on the contralateral shoulder
6. concomitant fracture of the shoulders/arms
7. concomitant neurovascular injury of the affected shoulder/arm
8. use of oral corticosteroids
9. age under 18 years or other reasons for lacking capability to consent.

### Obtaining informed consent

Patients presenting to the emergency department or outpatient clinic at any of the trial sites who show one of the included LHB tendon pathologies in MRI images will be assessed for eligibility. Patients who meet all eligibility criteria will be informed of the trial by the enrolling center’s investigators (LOOPTEN collaborators) and will receive written patient participation information. They will be given sufficient time to consider their decision. If consent to participate in the study has been obtained, a baseline assessment including epidemiologic patient data and baseline scores of all outcome parameters is scheduled (see Fig. 1 and Table 1).

**Table 1:** Timeline and overview of schedule of enrolment, interven﻿tions and assessments of LOOPTEN

	STUDY PERIOD						
	Pre-allocation		Allocation	Post-allocation			
TIMEPOINT	Patient screening	Study inclusion	Day 0	*6 weeks (± 7 days)*	*6 months (± 14 days)*	*12 months (± 30 days)*	*24 months (±30 days)*
ENROLMENT:							
Eligibility screen	X						
Informed consent		X					
Baseline data		X					
Allocation			X				
INTERVENTIONS:							
* Study Intervention (Implant-free loop tenodesis or Implant-based arthroscopic anchor tenodesis)*			X				
ASSESSMENTS:							
* LHB score*		X		X^a^	X	X	X
* Constant-Murley-score*		X		X^a^	X	X	X
* ASES score*		X		X	X	X	X
* DASH score*		X		X	X	X	X
* SF-36 questionnaire*		X		X	X	X	X
* Adverse events of special interest*			X	X	X	X	X
* Strength measurements*		X			X	X	X
* Ultrasound examination*		X		X	X	X	X
* MRI scan*	X^b^						X

^a^Strength measurements that are included in the CMS and ASES score are omitted at the 6-week follow-up

^b^Preoperative MRI is not a study-specific examination but is part of the clinical routine and is necessary to identify the pathologies of the LHB tendon and to enroll patients in the study

### Additional consent provisions for collection and use of participant data and biological specimens 

Not applicable since additional collection and use of participant data and biological specimens in ancillary studies is not planned.

## Interventions

### Intervention description 

Both surgical and perioperative treatment will be conducted by the enrolling center. The patient is placed in a standard position for arthroscopic surgery of the shoulder. After preparation of the surgical field using standard procedures in accordance with current recommendations [19], the required standard arthroscopic portals are created. Since both the experimental intervention and the control intervention can be performed using the same skin incisions, blinding of the patients to the treatment received is possible. The surgical procedure starts with diagnostic arthroscopy to confirm structural lesions of the LHB complex and concomitant pathologies. After diagnostic arthroscopy is finished, the randomized trial intervention of implant-free loop tenodesis (experimental intervention) or implant-based epiosseous anchor tenodesis (control intervention) is performed, regardless of the concomitant pathologies.

The study groups differ in the details on how the LHB tendon is reattached to a less stress-exposed area at the humeral head after the tendon is cut at its origin. While the control treatment uses an implant for secure fixation, the trial intervention does not require an implant:

1. *Experimental Intervention Group: Loop tenodesis technique*
  First, the intended position for tendon fixation at the entrance to the bicipital groove is debrided to create a slightly bleeding surface for improved tendon ingrowth. The LHB tendon is then securely grasped with a surgical clamp and cut close to its base. Afterwards, the tendon stump is pulled extracorporeally. About 5–15 mm of the injured proximal tenotomized tendon is then resected. The tendon “loop” is created extracorporeally by folding and doubling the proximal 1.5 cm of the LHB tendon stump in order to increase the proximal diameter of the tendon. The loop is fixed with a suture. After completion of the loop, the tendon is released and pushed intraarticularly. The tendon loop then locks into place at the previously debrided entrance to the bicipital groove, where it should gradually grow in.
2. *Standard of Care Intervention Group: Arthroscopic epiosseous anchor tenodesis technique*
  At first, the LHB tendon is fixed in the joint with a clamp to prevent retraction after cutting. Subsequently, the LHB is cut close to its origin using arthroscopic electrocautery. Next, the LHB tendon stump is pulled extracorporeally by the clamp. The diseased proximal part (5–15 mm) is then resected, and the tendon is armed using a Krackow stitch technique with a non-absorbable suture material. The LHB tendon is fixed using a bioabsorbable knotless bone anchor (SwiveLock®, Arthrex, Naples, FL, USA or comparable) that is inserted into the previously prepared proximal bicipital groove according to the manufacturer’s recommendations and attaches the suture armed tendon stump to the humeral head.

Any concomitant procedures [12] are performed after completion of the tenodesis procedure, depending on the presence of additional pathologies.

The standardized postoperative rehabilitation protocol is identical for both treatment groups. The supervised physical therapy program follows the available recommendations for arthroscopic tenodesis procedures by the international specialist societies. Patients are instructed to avoid all elbow flexion and supination maneuvers against resistance for 6 weeks. After 6 weeks, lightweight (500 g) active elbow flexion exercises are started. Intensified physical therapy (≥ 2x/week) is scheduled from week 7 to 12 with a gradual increase in load. Sports-specific training can be started after 12 weeks at the earliest, depending on individual mobility and coordination. Modifications of the postoperative rehabilitation protocol may be required depending on concomitant procedures.

### Explanation for the choice of comparators

Among the common techniques, the *arthroscopic suprapectoral epiosseous (onlay) implant-based tenodesis technique* is considered an appropriate comparator for the following reasons:

1. Since suprapectoral anchor tenodesis can be performed arthroscopically using the same standard arthroscopic portals that are also needed to perform the experimental intervention, the only difference from the experimental treatment will be the mode of tendon attachment (implant-based vs. implant-free), and implant-dependent outcome effects can be investigated without confounding factors such as additional skin incisions that would be needed for subpectoral tenodesis. The literature suggests that subpectoral tenodesis could carry a higher rate of perioperative surgical site infections [9].
2. Moreover, an additional skin incision for subpectoral biceps tenodesis would preclude blinding of the study patients. An additional wound would allow patients to recognize the treatment they had received.
3. The structural outcome of loop tenodesis should only be compared to techniques with comparable anchorage sites. In loop tenodesis, the tendon loop is positioned and later blocks itself at the entrance to the bicipital groove in a position comparable to arthroscopic anchor placement in the control group.
4. Since intraosseous tendon-to-bone-attachment with interference screws has been shown to have increased rates of Popeye deformity [20], only epiosseous techniques are considered appropriate as a control group.

### Criteria for discontinuing or modifying allocated interventions 

Not applicable since the allocated study interventions are completed by a one-stage surgical intervention.

### Strategies to improve adherence to intervention (standardized surgical treatment)

To ensure comparable and high-quality treatments across all centers, we plan to standardize surgical treatment by:

1. Pre-study surgeon training: Surgical techniques for both the experimental and the control interventions are already routinely performed at all participating centers. Nevertheless, educational visits of the principal investigators are planned at all centers to agree on the details of the surgical techniques using a published detailed step-by-step description and an instructional video [15] and to establish a common standard for both the surgical procedure and the standardized implementation of the examination methods to be carried out during the follow-up visits.
2. Documentation requirements: For appropriate documentation during the surgical procedures, a protocol has to be filled in that includes the study patient’s pseudonym, the tenodesis technique, the intraoperatively apparent LHB tendon pathology, the amount of proximal biceps tendon resection, and, if applicable, the implant used. Moreover, a standardized intraoperative image of the key step of each procedure should be taken and archived locally for each surgical intervention performed on study patients.

To standardize the assessments of outcomes, evaluation of the functional outcome as part of the clinical assessment will be done only by instructed and trained examiners to avoid high inter-rater variability. Strength measurements during follow-up visits will have to be performed with the same measuring devices at each study center.

### Relevant concomitant care permitted or prohibited during the trial 

The following additional treatments are permissible in the context of this study:

- Surgical treatments: If the LHB tendon is surgically treated as a primary indication, surgery is in most cases supported by auxiliary procedures [12] such as repair, augmentation, or debridement of the rotator cuff, subacromial debridement with/without distal clavicle resection, and labroligamentous procedures. These procedures are thus permissible in the context of the study, especially since the primary outcome parameter (LHB score) is highly specific for LHB-related symptoms and is not affected by these additional surgical procedures.
- Pain medication and other concomitant medication: all standard non-opioid (NSAR, metamizole, paracetamol) and opioid (oxycodone, hydromorphone, etc.) pain medication in demand-dependent dosing is permitted as there is no significant difference between these drugs in postoperative pain levels for soft tissue injuries in recent literature [21]. Since pain medication can usually be discontinued after at least 3 postoperative months, analgesics are not expected to have a significant effect on the primary endpoint of the study after 12 postoperative months. The use of corticosteroid drugs [22, 23] or fluoroquinolone antibiotics [24] is known to adversely affect tendon metabolism and healing and increase the risk of injury. The use of these active substances is permitted during the study period but must be precisely documented. Moreover, all other concomitant medication is permitted during study participation since no effect of any medication on the primary endpoint of the study is expected.
- Rehabilitation: Physical therapy is conducted postoperatively according to the standardized rehabilitation plan, which was designed in accordance with the recommendations of international specialist societies and approved in preparation for this trial by the German, Austrian, and Swiss Society for Shoulder and Elbow Surgery (DVSE) and our Loop Tenodesis Patient Committee. Depending on the performance of any additional procedure, modifications of the postoperative rehabilitation protocol may be required that include a prolonged resumption of exercise.

### Provisions for post-trial care 

Due the nature of the intervention and the general health status of the participants, no special post-trial care will be required. In the unlikely event of persistent study-related complications, any damages will be covered by the study insurance.

### Outcome assessment 

The follow-up examinations will be conducted by an independent examiner blinded to the tenodesis technique received by the study patient (observer-blinded).

#### Primary outcome measure

The *primary endpoint* will be the biceps-specific LHB score as assessed at 12 months after surgery. The score will also be recorded at baseline and at the 6-week, 6-month, and 24-month follow-ups. This highly disease-specific assessment of LHB-associated outcome after surgery includes patient-reported symptoms (pain, cramps, or tenderness), the cosmetic result (Popeye deformity), and an objectively measurable functional parameter (flexion strength), each compared to the healthy contralateral tendon [25], with patient-reported outcome weighted more heavily in the overall score than functional outcome [25, 26]. The LHB score has been shown in previous studies to be a valuable tool for evaluating outcome after LHB tenotomy and tenodesis procedures [17, 25–30].

#### Secondary outcome measures

*Assessment of safety:* Another part of the hypothesis of the trial is that implant-free loop tenodesis is associated with fewer implant-related complications. A core set of adverse events of special interest has been set up for this study (see Table 2).

While intraoperative complications will be assessed during the performance of the randomized procedure, postoperative adverse events of special interest will be specifically queried at each follow-up visit. Such events will be graded according to the modified Clavien-Dindo classification for orthopedic surgery [31], depending on the need for outpatient or surgical/inpatient treatment. Since the overall complication rate is expected to be low, as seen in previous trials [9, 12], differences in the complication rates were not considered a primary endpoint and were thus not included in the sample size calculation.

For the selection of *secondary outcome parameters*, we followed the suggestions for comprehensive patient assessment in clinical trials on shoulder surgery by Wright and Baumgarten [32] for the American Academy of Orthopedic Surgeons to combine general health outcome measures with general shoulder function measures and disease-specific shoulder measures. Since the disease-specific LHB Score was chosen as the primary outcome parameter, we added the following general health and general shoulder function measures as secondary outcome parameters:

- The Constant(-Murley) score and the ASES score (American Shoulder & Elbow Surgeons Society standardized Shoulder Assessment) were chosen for the assessment of global shoulder function because they are the first and second most frequently used shoulder function scores in the world [33]. Both scores will be part of each study visit.
- The disability of arm, shoulder, and hand (DASH) score serves as an evaluation tool of global function of the arm at each study visit. The score is recommended when more than one joint of the arm is affected, as is the case with pathologies of the biceps brachii.
- For the assessment of general health-related quality of life at each study visit, we chose the SF-36 questionnaire as another standard score in the related literature.
- Strength measurements: Elbow flexion strength is measured using an isometric dynamometer (IsoForce-Control®, Herkules Kunststoff AG, Oberburg, Switzerland) with the patient seated in a chair, 90 degrees of elbow flexion, and a supinated forearm. The patient is then instructed to pull on the loop attached to the isometric dynamometer. Moreover, forearm supination strength is examined using the Baseline hydraulic wrist dynamometer (Fabrication Enterprises Inc., White Plains, NY, USA) with the elbow immobilized in 90-degree flexion by a motion control splint. The patient is instructed to supinate the forearm with maximum force against a T-handle from a neutral position. All measurements are repeated three times, and a mean value is calculated. Both flexion and supination strength are assessed and compared to the unaffected contralateral arm as a secondary parameter. Strength assessment will start from the 6-month postoperative follow-up visit.
- B-mode ultrasound examination of the LHB tendon in the bicipital groove will be part of each follow-up visit to assess the structural integrity of the tenodesis construct at any time after surgery. It is performed in the transverse and longitudinal planes, and ultrasonographic images are locally stored for later comparison and measurement. To objectify this investigator-dependent diagnostic tool for later evaluation, the answering of several standardized binary questions (yes/no) will be mandatory.
- MRI examination is required to evaluate tendon ingrowth into the surrounding connective tissue at the attachment site at the final follow-up visit after 2 years, since MRI is considered the unbiased gold standard in tendon diagnostics.

### Participant timeline

The following baseline data will be collected for each study patient after written informed consent has been obtained: age, sex, height, weight, arm dominance, American Society of Anesthesiologists (ASA) grade, concomitant diagnoses and medications, previous arm surgery, and tobacco and alcohol use. All functional outcome scores will be assessed at enrollment prior to surgery to obtain preoperative scores.

After the allocated study intervention has been conducted, patients will be seen for follow-up examinations at four postoperative visits (after 6 weeks, 6 months, 12 months, and 24 months) to evaluate the study outcome parameters (see Table 1).

### Sample size calculation 

The primary objective of this study is to show the non-inferiority of loop tenodesis in comparison with arthroscopic anchor tenodesis with respect to the primary clinical outcome LHB score (scale range 0–100). The clinical non-inferiority margin was set at 5 points on the LHB score, which was based on statistical considerations and clinical judgment. Moreover, an expert panel of German-speaking shoulder surgeons was consulted on the study design and the choice of non-inferiority margin. The panel defined a non-inferiority margin of 5 points, taking into account the differences in LHB scores between the established procedures and the minimal clinically important differences in other related scores. For loop tenodesis to be considered standard of care and to show non-inferiority to implant-based tenodesis techniques, it has to outperform the technically easier procedure of biceps tenotomy. According to Kerschbaum et al. [26, 34], the mean LHB score of patients treated with biceps tenotomy is at least 5 points lower than that of patients treated with arthroscopic epiosseous suprapectoral anchor tenodesis. Setting the non-inferiority margin at 5 points can also be considered a benchmark for outperforming biceps tenotomy as a historical control, as suggested by studies by Kerschbaum et al. [26, 34]. Moreover, according to the literature [17, 25–28], we expect a mean LHB score of 85 with a standard deviation of 10 for both treatment groups at 12 months. The non-inferiority margin of 5 points is half of the expected standard deviation, which is widely accepted as the minimal clinically relevant difference of PROs [35]. To show non-inferiority with a non-inferiority margin of 5 points and a standard deviation of 10 points with a power of 90% at a one-sided 2.5% level of significance, a total of *n* = 172 patients are required for statistical analyses. Assuming an early drop-out rate of 10% (change of indication during arthroscopy and thus no need for surgery or no information on the clinical outcome (drop-out before week 6)), a total of *n* = 192 patients (96 per group) need to be randomized. Sample size was calculated by using SAS 9.4 and the procedure proc power.

### Recruitment 

Different measures were taken to minimize recruitment challenges and to improve adherence to interventions:

- The selected inclusion and exclusion criteria are not overly restrictive and therefore allow the recruitment of the majority of patients with the tendon pathologies of interest at the enrolling centers.
- There are no competing trials at the enrolling centers, so that all eligible patients can be enrolled in this trail without confounding factors. Since there is no seasonal fluctuation of the incidence of LHB pathologies, constant recruitment throughout the entire recruitment period is possible.
- To reduce the complexity of the protocol, we chose a simple two-arm, parallel group design, omitting a third control group treated with simple tenotomy. Within the control group, a single tenodesis technique was clearly defined as the appropriate comparator.

### Assignment of interventions: allocation 

Allocation to one of the two treatment arms in a 1:1 ratio will be randomized using block-wise randomization with different block sizes stratified by center. The randomization list and the block size will be concealed for all participants until the final endpoint has been analyzed. Randomization will be carried out using the clinical database management system (CDMS) managed by the Center for Clinical Studies at the University Hospital Regensburg. After a patient has been enrolled by signing the informed consent and on the day of surgery, the responsible personnel (investigator and/or study nurse) must use the individual login for the CDMS to randomize the patient. All randomizations will be logged and documented within the system, and predefined users such as the study monitor will be automatically informed about the randomization.

## Assignment of interventions: blinding

### Who will be blinded 

The trial includes two levels of blinding:

1. Trial participants will be blinded to their treatment allocation. This blinding is intended to reduce the risk of treatment-related behavior and patient awareness of treatment-related side effects. As the surgical treatment in both study groups can be performed using the same skin incisions and the implants in the control group are not visible in plain radiographs, patients will not be able to identify the treatment they have received (*patient-blinded*).
2. To avoid detection bias during the study visits, collection of the postoperative outcome parameters will be conducted by outcome assessors who are not aware of the treatment allocation and have not been included in the surgical procedures (*outcome assessor-blinded*).

### Procedure for unblinding if needed 

Unblinding is not necessary in any circumstances during study participation, since even in case of adverse events or need for revision surgery, continued blinding of both patients and outcome-assessors is feasible. Of course, unblinding of participants and study personnel is permissible, if patients withdraw consent to participate in the study.

## Data collection and management

### Data management strategy 

To facilitate the collection of study data, a web-based electronic case report form (eCRF) will be established within a clinical database management system (CDMS). This system will provide a comprehensive, auditable, and integrated environment that meets the regulatory requirements of ICH E6 (R2) and GDPR. Each investigator is responsible for reviewing and ensuring the accuracy, completeness, and timeliness of the pseudonymized patient data entered into the eCRF and will provide his/her signature and date of signature on the eCRF pages. During the study, monitors will review the eCRF entries by remote, and if necessary, by onsite source data verification in order to ensure accuracy, completeness, and plausibility of the data entered. Data entered into the study database will be systematically and periodically checked by senior data management staff for completeness, omissions, and values requiring further clarification, using computerized and manual procedures. In addition, a central statistical monitoring approach will be applied to improve data quality and site performance. Any errors or omissions are recorded on data query forms, which are forwarded to the study site for resolution. Quality control audits of all key safety and efficacy data in the database are made prior to locking the database. After study completion, all electronic study data will be converted into an auditable and system-independent accessible standard data format (CDISC) and stored for at least 10 years.

### Plans to promote participant retention and complete follow-up 

Different measures will be implemented to ensure participant retention:

- Study data may be collected as part of a brief follow-up visit that does not significantly exceed the scope of routine out-of-study follow-up. The time points of the follow-up visits are selected in concordance with the routine control visits in the outpatient clinic after surgery, so that participants are not overburdened with additional appointments for study purposes.
- To improve patient adherence, all follow-up appointments are scheduled at the end of the previous appointment, and patients will be reminded of the appointment by phone or email one week before the scheduled appointment. A 15-day window, defined as 7 days before and 7 days after the due date, will be available to complete the 6-week and 29-day windows, defined as 14 days before and 14 days after the due date for the 6-month follow-up assessments and a 61-day window, defined as 30 days before and 30 days after the due date, will be available to complete the 12-month and 24-month follow-up assessments.

### Confidentiality 

See section on data management above.

### Plans for collection, laboratory evaluation and storage of biological specimens for genetic or molecular analysis in this trial/future use 

Not applicable, since no collection, laboratory evaluation, and storage of biological specimens is planned.

### Statistical methods 

Primary estimand: Within the population of all randomized patients defined by the inclusion and exclusion criteria with at least one assessment of the LHB total score at any point in time, the primary endpoint of LHB total score at 12 months after surgery will be analyzed. The occurrence of the intercurrent events such as reoperation procedures, additional operations, or further injuries of the ipsi- or contralateral distal or proximal biceps tendon will be disregarded (treatment policy strategy). We expect a maximum of 10% of patients to experience an intercurrent event within the first 12 months after surgery. The summary measure will include the following methods: Point estimates for both treatment arms will be presented as mean and standard deviation with corresponding 95% confidence interval. To test the null hypothesis H_0_: μ_anchor_ – μ_loop_ ≥ 5 against the alternative hypothesis H_1_: μ_anchor_ – μ_loop_ < 5 (μ_anchor_, μ_loop_: mean of LHB total score after arthroscopic anchor tenodesis, loop tenodesis at month 12) at a one-sided significance level of 0.025, a generalized linear model (GLM) with the LHB score at month 12 as the dependent variable and the type of treatment and center as fixed factors and LHB score at baseline as covariate will be used. The difference between anchor tenodesis and loop tenodesis will be presented as the least squared means together with an associated 97.5% one-sided confidence interval based on the GLM. If the upper bound of the CI is not higher than 5 points, then the null hypothesis will be rejected. If non-inferiority of loop tenodesis is determined, superiority of loop tenodesis will then be tested in the same way. With an expectation of no more than 10% missing values for the LHB score at month 12, which will be considered missing completely at random (MCAR) or missing at random (MAR), multiple imputation using the Markov chain Monte Carlo (MCMC) method will be used to account for such missing values. The MCMC imputation model will include the LHB measures at 6 weeks, 6 months, and, if available, at 24 months, as well as the baseline patient characteristics age, sex, BMI, dominant hand, and further injuries. A sensitivity analysis using a GLM on ranks will be used to explore the robustness of inference from the initial model.

The 2^nd^ estimand is based on the composite policy strategy. The endpoint is the LHB total score > 80 points at 12 months after surgery as a binary endpoint. Intercurrent events as defined above will be classified as non-responders regardless of the LHB score at month 12. The summary measure will be the difference in the proportions between the two treatment arms.

The 3^rd^ estimand is based on the hypothetical policy strategy. The endpoint and the summary measure are defined according to the primary estimand, but for all patients with intercurrent events, the LHB score at month 12 will be replaced by a hypothetical score. This score is based on a multiple imputation method as for the primary estimand, using only information before the occurrence of the intercurrent event. All estimands together will provide a good answer to the question of whether the new method of loop tenodesis is non-inferior or even superior to the standard anchor tenodesis procedure. Furthermore, subgroup analyses based on age and sex will be performed. Statistical analyses of the secondary endpoints will be carried out in an exploratory manner without any multiplicity adjustments as described in detail in the Statistical Analysis Plan. Descriptive safety analyses will be provided.

### Interim analyses 

Not applicable, since no interim analyses are planned.

### Plans to give access to the full protocol, participant level-data and statistical code 

The statistical analysis plan will be made available as an addendum to the primary paper. Individual deidentified participant data (including data dictionaries) will be shared through Zenodo, a European open access data repository. Data and documents will be made available to interested researchers upon a reasonable request for a period of five years.

(See section on dissemination plan below).

## Oversight and monitoring

### Composition of the coordinating center the data safety and monitoring board (DSMB), and the trial steering committee 

The trial will mainly be managed by two clinical trial coordinators, who are also the principal investigators of the trial, and one additional project manager, all of whom are members of the Department of Trauma Surgery at the University Hospital Regensburg. Randomization, data management, and biometric analysis will be done by the Center for Clinical Studies (ZKS) at the University Hospital Regensburg. Each recruiting center will be represented by a cooperating investigator, who is responsible for the coordination and performance of local recruitment, randomization, patient-blinded treatment, and observer-blinded follow-up examinations. To ensure adherence to the intervention scheme and the quality of performance of each recruiting center, an independent Data Safety and Monitoring Board (DSMB) has been established, consisting of three experienced shoulder surgeons and researchers who are not involved in the conduct or design of the trial and are not part of the medical institutions involved. The Board's responsibility will be verifying the proper conduct of the study with respect to randomization, blinding, and quality of surgical techniques using the mandatory documentation during online meetings following each on-site monitoring visit by the independent study monitor. Furthermore, the Board will advise on the continuation, modification, or termination of the whole trial in the event of cumulative occurrence of adverse events of special interest and failure to meet recruitment milestones as part of the meetings. Moreover, the chairman of the Commission for Research and Development of the German, Austrian, and Swiss Societies for Shoulder and Elbow Surgery (DVSE Kommission “Research und Development”) has agreed to assume the patronage of the trial and supervise the overall progress of the study and the publication of the results. In case of structural, coordination, or organizational problems, he will advise the recruiting centers.

### Composition of the data monitoring committee, its role and reporting structure 

Quality assurance will consist of a combination of remote monitoring and on-site monitoring. Remote monitoring will be done by the data manager and will focus on data flow and accuracy in completing the eCRF. If performance falls below a predefined quality threshold, additional on-site monitoring visits will be scheduled. On-site monitoring will be commissioned to the CRO multi-service-monitoring, which has specialized in the monitoring of non-commercial IITs since 2000. The monitors are qualified according to ICH-GCP and DIN ISO 14155 and follow the CRO’s SOPs MON 002, 003, 007, and 008. On-site monitoring starts with a pre-trial visit to each center in order to ensure that each center is capable of complying with the study protocol and recruiting an adequate number of patients. The findings of the pre-trial monitoring visit will be summarized in a report, which will be forwarded to the PI and the steering committee. The monitoring will follow a risk-based approach, and the study will be considered a low-risk trial. Thus, 100% source data verification focuses on informed consent, inclusion/exclusion criteria, adverse events of special interest, the primary endpoint, and randomization. All other aspects of the trial are subject to a 20% source data verification. In addition to the pre-trial visit, on-site monitoring is scheduled at the beginning (initiation visit) and at the end of the study (closeout visit), and at months 6, 12, and 24.

### Stopping rules

- For patients: As guaranteed prior to study inclusion, the individual patient will be excluded from study participation if the patient withdraws his or her informed consent. Other stopping rules for patients are not applicable due to the single-day character of the trial intervention.
- For participating centers: Recruitment and protocol-compliant implementation of the follow-up examinations will be monitored by the DSMB throughout the entire trial period. If recruitment at the 50% landmark is less than 20% of the total target number of patients, the enrolling center will have to be excluded from participating in the trial.
- For the entire trial: If recruitment in more than one center is not possible, the Trial Steering Committee will be consulted to determine whether continuation of the trial is still realistic. If the incidence of adverse events of special interest with the trial intervention is significantly higher than that in the control group (> 50% more complications), continuation of the trial must be discussed with the DSMB and the Trial Steering Committee.

### Adverse event reporting and harms 

The occurrence of surgery-related adverse events of special interest (see Table 2) will be collected by each treating center throughout the follow-up period and recorded centrally at the Center for Clinical Studies (ZKS) of the University Hospital Regensburg. The safety assessments will be summarized by the statistician, and safety reports will be forwarded to the DSMB. In case of the occurrence of serious incidents, the enrolling center notifies the Federal Institute for Drugs and Medical Devices (BfARM).

**Table 2:** Adverse events of special interest that will be monitored both intraoperatively (left) and postoperatively at each follow-up examination (right)

Intraoperative events	Postoperative events
Implant breakage/pullout	Displacement (Anchor migration/pullout, any change of the position of the tendon anchorage compared with previously documented position)
Implant malposition	Fracture of humeral head
Suture knot slippage/breakage	Bone cyst/osteolysis
Tendon slippage into the groove	Surgical site infection (superficial/deep)
Fracture	

### Frequency and plans for auditing trial conduct 

See section on monitoring above.

### Plans for communicating important protocol amendments to relevant parties

There will be a study newsletter administered by the coordinating investigator and the Center of Clinical Studies at the University Hospital Regensburg, which will report study progress and amendments to the principal investigator and the cooperating investigators at each center at a regular interval. In case of changes of the study protocol, the coordinating investigators will inform and obtain the further approval of the Ethics Committee.

### Dissemination plans

The data collected during this study are confidential and property of the sponsor and will be presented at international meetings and conferences. Data from this study will be published open-access in a peer-reviewed journal. The statistical analysis plan will be made available as an addendum to the primary paper. Individual deidentified participant data (including data dictionaries) will be shared through Zenodo, a European open access data repository. Data and documents will be made available to interested researchers upon a reasonable request for a period of five years.

## Discussion

Gartsman et al. have shown that shoulder disorders reduce patients'self-assessed quality of life to a similar extent as the five major medical conditions (hypertension, congestive heart failure, acute myocardial infarction, diabetes mellitus, and clinical depression) [36]. Shoulder arthroscopy is the fourth most common orthopedic procedure in the US [2]. In the adult patient population, shoulder pain is commonly associated with pathologies of the LHB tendon [1]. For the arthroscopic treatment of these pathologies, Walch et al. first proposed simply cutting the tendon without additional reattachment [37] as an alternative to the then standard treatment of open tenodesis. Walch et al. hypothesized that, after tenotomy, the LHB tendon blocks in the lower bicipital sulcus due to its physiologically wider diameter at the proximal tendon origin and later “auto-tenodeses” by scar tissue formation. This hypothesis of autotenodesis could later be confirmed in animal trials [38] and in clinical studies [39–41]. Later, when arthroscopic tenodesis procedures using suture anchors were developed, these procedures showed improved functional outcomes, supination strength, and lower rates of cosmetic deformity when compared to arthroscopic tenotomy in large trials and meta-analyses [6–8]. However, for safe refixation of the tendon, these techniques all use some form of implant, which carries the risk of implant-associated adverse events [11–14, 42, 43]. To avoid these risks by omitting a bone anchor, a new easy-to-learn and quick-to-perform arthroscopic technique was developed by Kerschbaum in 2019 [15], which provides stable attachment of the tendon to the bone without the need for an implant. The rationale behind this loop tenodesis procedure is as follows: In contrast to all other currently accepted standard tenodesis techniques, which all use some type of implant for primary tendon-to-bone attachment, the new tenodesis technique provides stable tendon anchorage without an implant by enlarging the proximal tendon diameter by doubling the tendon stump and, therefore, improving the autotenodesis mechanism. The new procedure is meant to combine secure attachment of the tendon comparable to established tenodesis techniques with ease of performance and a low complication rate comparable to tenotomy. After biomechanical testing, loop tenodesis has shown significantly higher failure loads than tenotomy [16]. A prospective case series recorded a decrease in biceps-related complaints and improved shoulder function [17] comparable to historical anchor tenodesis cohorts [27, 34]. Subsequently, the present study aims to provide much-needed robust evidence to establish this relatively new implant-free procedure versus a comparable implant-based arthroscopic tenodesis procedure.

Thus, the two techniques investigated in this trial differ in the way in which the LHB tendon is attached to the humeral bone (implant-based vs. implant-free) and is secured against slippage through the bicipital groove. By participating in the current trial, patients receive blinded treatment for LHB tendon pathologies. Although the experimental treatment group will be treated with a new surgical technique with little data available, we expect a high willingness to participate in the study because the details of the tenodesis technique usually play a subordinate role in patient perception and both the control intervention and the experimental intervention are already routinely performed surgical techniques in daily practice. Nevertheless, the recruitment plan requires patients to be enrolled regardless of concomitant pathologies and concomitant arthroscopic procedures. This is unavoidable because isolated pathology of the LHB tendon without accompanying shoulder pathologies accounts for only 5% of cases and is, therefore, a rarity in everyday clinical practice [44]. The sole inclusion of patients with isolated pathologies of the long biceps tendon would therefore not only question the feasibility of the recruitment target based on the sample size calculation, but would also not represent the reality of clinical practice. To overcome the limitation of heterogeneity of the study population and selection bias of the results, the biceps-specific LHB score was chosen as a primary outcome parameter for this trial. The score was introduced in 2011 [25] in order to provide a useful tool for the highly specific evaluation of the LHB tendon after surgery, because the most established shoulder scores only incompletely cover LHB tendon disorders and mainly depend on the results of concomitant procedures. The LHB score has proven to be a valuable tool for evaluating outcome after LHB tenotomy and tenodesis procedures in previous studies [17, 25–30].

Moreover, the prospective cohort study on the short-term outcome after loop tenodesis is a good indication that the targeted recruitment numbers are achievable. In the pilot study on mid-term outcomes after loop tenodesis, which was conducted as a single-center study in one of the recruiting institutes (Sporthopaedicum Regensburg), the same inclusion and exclusion criteria were applied. It was possible to enroll 55 patients within 7 months [17]. If the number of patients included in the pilot study is extrapolated to the recruitment period of 12 months as planned in the current RCT, the single reference center alone will be able to enroll 94 patients.

The study will provide high-evidence data for a new technique in the field of surgical treatment of the long biceps that is expected to provide the following benefits: non-inferior biceps function and cosmesis compared to current standard-of-care treatment and low complication rates.

## Trial status

This is the first version of this protocol (August 2024). Recruitment of participants will start in December 2024 and is expected to conclude in November 2025. All patients will be followed up for 24 months. The final results are expected to be available after in December 2027 and disseminated via peer review publication and conference presentations.

## Data Availability

No datasets were generated or analysed during the current study.

## Abbreviations

AAT: Arthroscopic anchor tenodesis
ASES: American Shoulder & Elbow Surgeons Society
CDMS: Clinical database management system
CMS: Constant-Murley-Score
CRO: Contract Research Organization
DASH: Disabilities of Arm, Shoulder, and Hand (questionnaire)
DRKS: German Register of Clinical Trials (*Deutsches Register Klinischer Studien*)
DSMB: Data Safety and Monitoring Board
DVSE: German, Austrian and Swiss Society for Shoulder and Elbow Surgery *(D.A.CH.-Vereinigung für Schulter und Ellenbogenchirurgie)*
eCRF: Electronic case report form
GLM: Generalized mixed model
ICH-GCP: International Council for Harmonization – Good Clinical Practice Guideline
LHB: Long Head of Biceps
LT: Loop Tenodesis
MCMC: Markov chain Monte Carlo
MRI: Magnetic Resonance Imaging
PI: Principal investigator
PRO: Patient-reported outcome
RCT: Randomized Controlled Trial
SF-36: Short Form - 36
SOP: Standard operating protocol
SPIRIT: Standard Protocol Items: Recommendations for Interventional Trials
ZKS: Center for Clinical Trials (*Zentrum für Klinische Studien*)
